# Risk of severe outcomes among SARS-CoV-2 Omicron BA.4 and BA.5 cases compared to BA.2 cases in England

**DOI:** 10.1016/j.jinf.2023.04.015

**Published:** 2023-04-24

**Authors:** Nurin Abdul Aziz, Sophie Grace Nash, Asad Zaidi, Tommy Nyberg, Natalie Groves, Russell Hope, Jamie Lopez Bernal, Gavin Dabrera, Simon Thelwall

**Affiliations:** UKHSA COVID-19 Vaccines and Epidemiology Division; UKHSA COVID-19 Vaccines and Epidemiology Division; UKHSA COVID-19 Vaccines and Epidemiology Division; MRC Biostatistics Unit, University of Cambridge, Cambridge, UK; UKHSA Genomics and Public Health Analysis; UKHSA HCAI, Fungal, AMR, AMU & Sepsis Division; UKHSA COVID-19 Surveillance Cell; NIHR Health Protection Research Unit for Respiratory Infections; UKHSA COVID-19 Vaccines and Epidemiology Division; UKHSA COVID-19 Vaccines and Epidemiology Division

**Keywords:** SARS-CoV-2, Severity, COVID-19 variants, Omicron BA.4, Omicron BA.5

Dear Editor,

A recent study by Kang and colleagues (1) found that individuals infected with the SARS-CoV-2 BA.5 Omicron sub-lineage exhibited more severe symptoms at the onset of symptomatic disease but had a shorter viable virus shedding period compared to individuals with BA.1 or BA.2. However, the study did not elucidate whether these differences reflected any variability in severe outcomes as the disease progressed. Additionally, data on BA.4 severity compared to previous sub-lineages are limited.

Studies have shown that both BA.4 and BA.5 have a spike protein mutation with the potential for immune evasion (2), which may result in increased severity for BA.4 and BA.5 compared to previous sub-lineages. Herein, we used a case-control study to assess relative severity of BA.4 and BA.5 compared to BA.2 by estimating the differences in risk of severe outcomes following presentation to emergency care. While this has been undertaken in other countries with similar demographics, these studies have been conducted in settings with low vaccine coverage.

The study included individuals with a PCR confirmed SARS-CoV-2 infection (COVID-19) in England between 1 April 2022 and 1 August 2022 inclusive, where linked whole genome sequencing (WGS) results were available confirming infection with the sub-lineages BA.2, BA.4, or BA.5, and who had attended an emergency department (ED) between one day before their positive test date and 14 days after their positive test date.

Two case-control outcome definitions were considered. Outcome definition 1 included individuals whose ED attendance ended in hospital admission or transfer with a length of stay in hospital of 2 or more days; or whose ED attendance ended in death. They were compared with a control group of individuals whose ED attendance ended with discharge or a hospital admission of less than 2 days’ duration, and who did not die in the 2 days following ED attendance.

Outcome definition 2 included COVID-19 patients who attended ED and received oxygen therapy. Controls were those who attended ED but did not receive oxygen therapy. This provided validation for definition 1 and a detailed metric for severity by using oxygen therapy as an indicator, as oxygen therapy has been frequently used in critical cases of COVID-19 (3).

Odds ratios (OR) of the outcomes and 95% confidence intervals (CI) were estimated using conditional logistic regression models. The models were stratified for week of positive test, and adjusted for age, sex, vaccination status, prior infection status, socioeconomic deprivation, region of residence, and ED attendances during the July extreme heat event. Further information on data acquisition and statistical analysis can be found in the supplementary document.

A total of 23,023 individuals were included in the study. The number of BA.2, BA.4, and BA.5 patients included in the study reflected the temporal trends in overall COVID-19 test-positive incidence during the study period (Figure 1). 21,725 patients met the criteria to be included in the analysis according to outcome definition 1. Across all variants, those admitted or who died were more likely to be older.

Higher proportions of BA.4 and BA.5 patients had received a spring booster or more than one booster, compared to BA.2 patients.

Using definition 1, the crude odds of admission or death after ED attendance for BA.4 (OR: 0.70; 95% CI: 0.63-0.77) and BA.5 (OR: 0.82; 95% CI: 0.77-0.86) were lower compared to BA.2 (Table 1). However, after adjustment, the difference in the odds of admission or death was not statistically significant for either BA.4 (OR: 0.96; 95% CI: 0.86-1.08) or BA.5 (OR: 1.02; 95% CI: 0.93-1.12) compared to BA.2. For outcome definition 2, the OR estimates were similar but had wider confidence intervals due to a smaller sample size. Sensitivity analyses exploring alternative inclusion criteria, study periods, and outcome definitions provided consistent results (supplementary document).

Our results do not suggest a difference in the risk of hospital admission or death, or in oxygen supplementation, following presentation to ED for BA.4 or BA.5 patients compared to BA.2 patients. These results are consistent with evidence for minor differences in risk based on other cohorts of community tested COVID-19 patients (4–6). However, they differ from a community testing cohort in Denmark which found greater odds of hospitalisation for BA.5 compared to BA.2 (7). The reasons for the discrepancies are unclear, and further research is warranted on the severity between Omicron sub-lineages in additional settings.

COVID-19 testing policy in England changed on 01 April 2022, resulting in a reduction in testing overall and a change in the selection of specimens for WGS, which targets high-risk groups within the population (8). This meant that previous cohort approaches to estimate relative severity of SARS-CoV-2 variants (9,10) were no longer feasible. To resolve this problem, we conducted a case-control study of the risk of severe outcomes for those presenting to ED. Samples taken in hospital are preferentially sequenced; consequently, this population may reflect the subgroup of SARS-CoV-2-infected individuals at risk of hospital admission, as ED is the primary route by which people with severe COVID-19 would be admitted to hospital.

However, this approach presents its own limitations. The probability of presenting to emergency care is a function of the severity of infection. Thus, people who are classified as controls are likely to be those for whom the infection is more severe than infections experienced in the general population. This restriction to COVID-19 patients with relatively severe disease regardless of variant might lead to biased estimates of relative risks compared to those that would be estimated if data derived from community mass testing were available.

While recent dominant Omicron sub-lineages in England have shown no increase in severity relative to the previous dominant variant (9), it cannot be assumed that future lineages will continue this trend. Hence, continued surveillance of severe outcomes of novel SARS-CoV-2 variants is warranted. The methodology used in this study will be useful to monitor severity of COVID-19 among individuals who experience sufficiently severe disease to seek emergency care during periods with limited availability of testing; this will be vital to inform the public health response to future emerging variants.

## Supplementary Material

Supplementary Materials

## Figures and Tables

**Figure 1:**
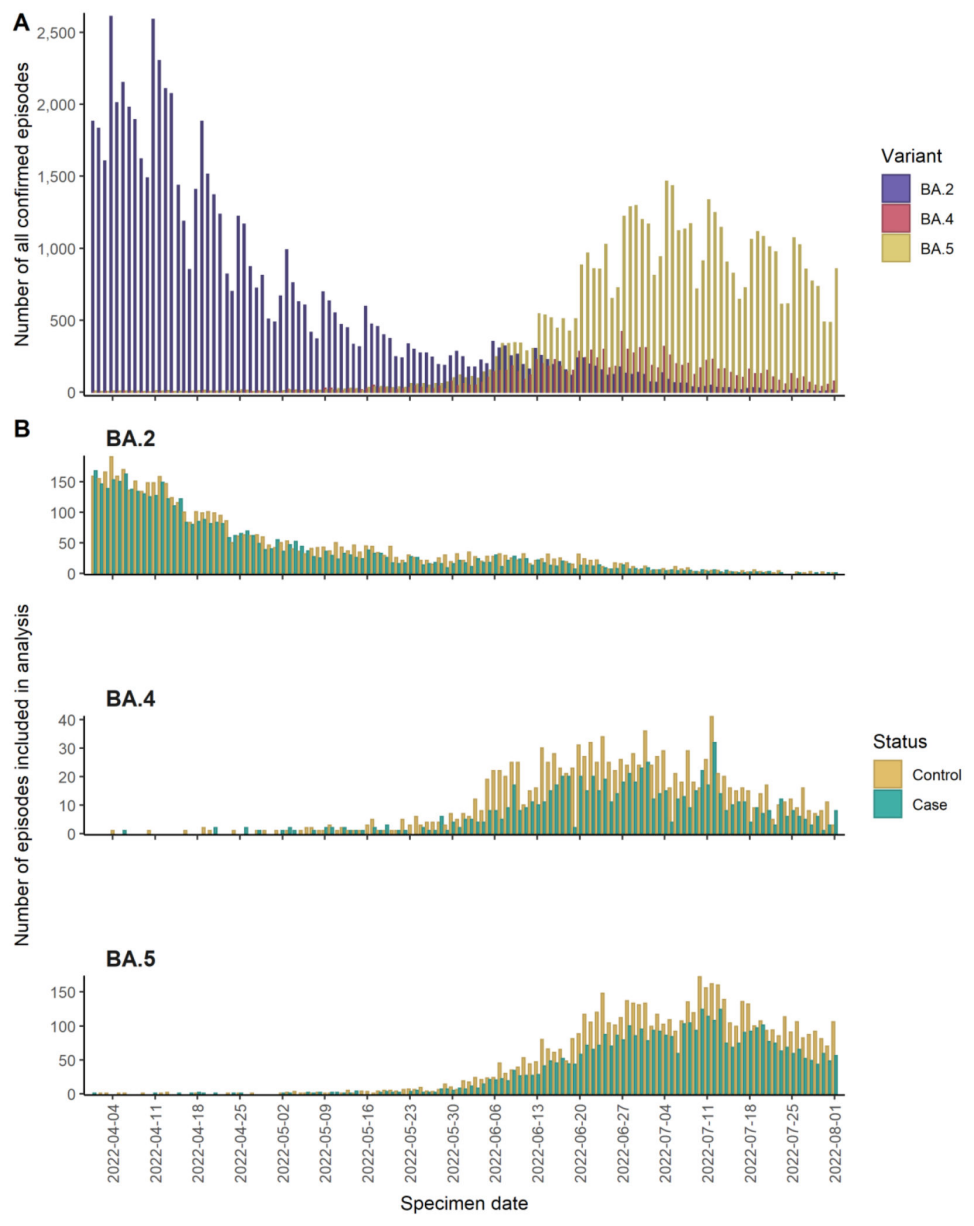
(A) Incidence of all confirmed COVID-19 Omicron BA.2, BA.4, and BA.5 in England between 1 April 2022 – 1 August 2022. (B) Incidence of individuals with BA.2, BA.4, and BA.5 included in the analysis by outcome status, according to outcome definition 1 where cases are individuals admitted ≥2 days or died within 2 days of attending ED and controls are individuals discharged from ED or admitted for <2 days.

**Table 1:** Crude and adjusted odds ratios comparing risk of admission or death among individuals who attended A&E for COVID-19 cases with Omicron BA.4 and BA.5 compared to Omicron BA.2, by outcome definition. Data includes positive tests in England between April – August 2022.

	BA.4 versus BA.2				BA.5 versus BA.2			
Case Definition	Crude Model		Adjusted Stratified Model*		Crude Model		Adjusted Stratified Model	
	OR (95% CI)	P-value	OR (95% CI)	P-value	OR (95% CI)	P-value	OR (95% CI)	P-value
Definition 1^†^	0.7 (0.63 - 0.77)	< 0.0001	0.97 (0.86 - 1.08)	0.5297	0.82 (0.77 - 0.86)	< 0.0001	1.01 (0.93 - 1.11)	0.7678
Definition 2^‡^	0.76 (0.62 - 0.94)	0.0107	0.94 (0.70 - 1.28)	0.7118	0.89 (0.80 – 1.00)	0.0483	1.18 (0.92 - 1.53)	0.1847

* Model is a conditional logistic regression stratified by specimen test week, and adjusted for age group (10-year age bands), vaccination status, sex, prior infection status, and a flag for possible heatwave-related hospital attendances.

† Definition 1: A case is any individual whose ED attendance ended in hospital admission with a length of stay in hospital of 2 or more days; if their ED attendance ended in death; or if they had a date of death up to 2 days after their initial date of ED attendance. A control is any individual whose ED attendance ended with discharge or a hospital admission of less than 2 days’ duration, and absence of death in the 2 days following ED attendance.

‡ Definition 2: A case is any individual who attended ED and received oxygen therapy in ED. A control is any individual who attended ED but did not receive oxygen therapy.

## Data Availability

The individual-level nature of the data used risks individuals being identified, or being able to self-identify, if the data are released publicly. Requests for access to these non-publicly available data should be directed to UKHSA.

## Highlights

- Immune evasion in Omicron BA.4 and BA.5 may increase risk of severe COVID-19
- BA.4/BA.5 severity was compared against BA.2 among patients attending hospital
- There was no difference in severity in patients with BA.4/BA.5 relative to BA.2
- Hospital-based case-control methods can be used to assess severity with limited testing
