# Supplementary material for: Risk of severe outcomes among SARS-CoV-2 Omicron BA.4 and BA.5 cases compared to BA.2 cases in England
Source: J Infect. Author manuscript; Available in PMC 2023 Jul 1. (PMC10124096; doi:10.1016/j.jinf.2023.04.015)
Supplement: Supplementary Materials [file EMS175016-supplement-Supplementary_Materials.pdf]

Risk of severe outcomes among SARS-CoV-2 Omicron BA.4 and BA.5 cases compared to BA.2 cases in England

Supplementary Materials

Nurin Abdul Aziz[1], Sophie Grace Nash[1], Asad Zaidi[1], Tommy Nyberg[2], Natalie Groves[3], Russell Hope[4], Jamie Lopez Bernal[5,6], Gavin Dabrera[1], Simon Thelwall[1]

[1]UKHSA COVID-19 Vaccines and Epidemiology Division

[2]MRC Biostatistics Unit, University of Cambridge

[3]UKHSA Genomics and Public Health Analysis

[4]UKHSA HCAI, Fungal, AMR, AMU & Sepsis Division

[5]UKHSA COVID-19 Surveillance Cell

[6]NIHR Health Protection Research Unit for Respiratory Infections

Supplementary Methods

Study design

The case-control study included individuals of all ages with a PCR confirmed SARS-CoV-2 infection in England between 1 April 2022 and 1 August 2022 inclusive, for whom linked WGS results were available for the same episode of infection[1], and who had attended an emergency department (ED). Individuals were included if they had attended ED between one day before their positive test date and 14 days after their positive test date. Data was extracted on 27 September 2022.

The study was restricted to individuals with WGS results confirming infection with the Omicron sub-lineages BA.2, BA.4, or BA.5. Individuals with descendant lineages BA.2.75 and BA.4.6 were retrospectively identified and excluded as they are considered distinct variants for the purposes of public health surveillance.

Individuals were also excluded if covariate information on age, sex, vaccination status, index of multiple deprivation (IMD) quintile, and region of residence were missing. Healthcare workers identified via the SARS-CoV-2 immunity and reinfection evaluation (SIREN) study, which involves regular screening of healthcare workers[2], were excluded as their screening test results are not representative of infection in the wider population eligible for symptomatic PCR testing.

Data sources

Individual-level data on laboratory-confirmed SARS-CoV-2 infections were extracted from the Second Generation Surveillance System (SGSS), the national surveillance system that holds test results from diagnostic laboratories in England for infectious diseases[3]. To ascertain variant type, patients were linked to validated WGS data coordinated by the COVID-19 Genomics UK consortium and made available in the Cloud Infrastructure for Big Data Microbial Bioinformatics database[4].

COVID-19 data were then linked to the Emergency Care Data Set (ECDS) and the Secondary Uses Service (SUS) dataset to obtain data on hospital attendance and admission, including SNOMED codes derived from the ECDS dataset. This includes data submitted by National Health Service (NHS) trusts up to 27 September 2022[5]. Vaccination status was determined using data from the

[1] UK Health Security Agency, 'Cases Definition to Include Multiple Infection Episodes from 31 January 2022', Coronavirus in the UK, accessed 30 December 2022, https://coronavirus.data.gov.uk/details/whats-new/record/af008739-ffa3-47b8-8efc-ef109f2cfbdd.
[2] UK Health Security Agency, 'SIREN Study', GOV.UK, accessed 30 December 2022, https://www.gov.uk/guidance/siren-study.
[3] Tom Clare et al., 'Timeliness and Completeness of Laboratory-Based Surveillance of COVID-19 Cases in England', *Public Health*, April 2021, S0033350621001219, https://doi.org/10.1016/j.puhe.2021.03.012.
[4] Thomas R. Connor et al., 'CLIMB (the Cloud Infrastructure for Microbial Bioinformatics): An Online Resource for the Medical Microbiology Community', *Microbial Genomics* 2, no. 9 (September 2016): e000086, https://doi.org/10.1099/mgen.0.000086.
[5] Alex Bhattacharya et al., 'Healthcare-Associated COVID-19 in England: A National Data Linkage Study', *The Journal of Infection* 83, no. 5 (November 2021): 565–72, https://doi.org/10.1016/j.jinf.2021.08.039.

National Immunisation Management Service (NIMS)[6]. Data on COVID-19 deaths was sourced from the UKHSA COVID-19 mortality dataset[7].

Variant definitions and lineage assignment

The sequence data used in this analysis is classified using UKHSA variant definitions and not using Pangolin[8] lineage assignment, which is commonly used to assign lineages to sequences. Pangolin uses mutations present throughout the genome to place sequences in a phylogeny and assign a lineage. UKHSA defines variants based on a set of mutations common to a lineage that can be used in combination to consistently detect, monitor, and report cases that share infection with a lineage. Lineage BA.4 was declared variant V-22APR-03 and BA.5 was declared variant V-22APR-04 on 6 April 2022[9].

The definitions include a set of mutations relevant to the Wuhan-1 reference (NC_045512) that can be used in combination to distinguish the variant from other SARS-CoV-2 variants. A minimum number of mutations required to provide sufficient sensitivity and specificity is determined. The list of mutations and calling requirements can be found on the UKHSA variant definitions repository[10]. Any sequences that do not meet these criteria will not be reported in the variant counts regardless of whether there is other evidence to suggest that the sequence belongs to the relevant pangolin lineage. Sequences that do not meet the current UKHSA definition but are assigned by pangolin to a relevant lineage are reviewed to ensure the suitability of these variant definitions over time.

Outcome and adjustment variable definitions

Two possible case-control outcome definitions were considered. Outcome definition 1 included individuals whose ED attendance ended in hospital admission or transfer with a length of stay in hospital of 2 or more days; or whose ED attendance ended in death; or who died up to 2 days after their initial date of ED attendance. They were compared with a control group of the COVID-19 patients whose ED attendance ended with discharge or a hospital admission of less than 2 days' duration, and who did not die in the 2 days following ED attendance.

---

[6] NHS England, 'National Vaccination Programmes', NHS England, accessed 29 September 2022, https://www.england.nhs.uk/contact-us/privacy-notice/national-flu-vaccination-programme/#immunisation.

[7] Alison E. Brown et al., 'Epidemiology of Confirmed COVID-19 Deaths in Adults, England, March–December 2020', *Emerging Infectious Diseases* 27, no. 5 (May 2021): 1468–71, https://doi.org/10.3201/eid2705.203524.

[8] Áine O'Toole et al., 'Assignment of Epidemiological Lineages in an Emerging Pandemic Using the Pangolin Tool', *Virus Evolution* 7, no. 2 (1 December 2021): veab064, https://doi.org/10.1093/ve/veab064.

[9] UK Health Security Agency, 'SARS-CoV-2 Variants of Concern and Variants under Investigation: Technical Briefing 40', 8 April 2022.

[10] Matt Bull et al., *Standardised Variant Definitions* (2021; repr., phe-genomics, 2022), https://github.com/phe-genomics/variant_definitions.

Outcome definition 2 included COVID-19 patients who attended ED and received oxygen therapy in ED according to the SNOMED code (57485005) provided in the hospital data. Controls were COVID-19 patients who attended ED but did not receive oxygen therapy.

Vaccination status was defined as the number of vaccine doses and boosters received at least 14 days before an individual's positive test. A booster is defined as an additional dose of vaccine given after the primary course of two doses to maintain strong protection against severe COVID-19 disease; this is separate from a third additional dose, which is administered to clinically vulnerable individuals to improve their response to the primary course[11]. This analysis included individuals who only received the primary course (one or two doses); those who received the primary course (including those with a third additional dose) and a first early booster, delivered in September 2021[12]; and those who received the primary course (including those with a third additional dose) and a seasonal spring booster, delivered in March 202[13], or both available boosters. Those with three primary doses and no boosters are included among those who have had their primary course and an early booster.

Extreme heat event attendances were defined as attendances where the arrival date was 19 or 20 July 2022, or attendances where the first or second day of stay in hospital fell on the same dates. This reflects attendances which may have been related to the extreme heat event on 18 and 19 July 2022 in England[14].

Statistical analysis

Analyses were run separately for individuals with BA.4 or BA.5 against BA.2. Odds ratios (OR) of the outcomes and 95% confidence intervals (CI) were estimated using conditional logistic regression models. The models were stratified for week of positive test, and further adjusted for age using categorical 10-year age bands, sex, vaccination status, prior infection status, IMD quintile (measure of area-level socioeconomic deprivation), NHS region of residence, and attendances to A&E during the July extreme heat event. To validate the results of the main analysis using outcome definition 1, crude and adjusted models were also run using outcome definition 2.

While access to free testing for COVID-19 was removed for the general population, people considered to be at high risk due to pre-existing conditions were provided with free tests in order to access antiviral or monoclonal therapeutics[15]. As a result, people who present to emergency care with

---

[11] 'Booster Shots and Additional Doses for COVID-19 Vaccines — What You Need to Know', 9 November 2022, https://www.hopkinsmedicine.org/health/conditions-and-diseases/coronavirus/booster-shots-and-third-doses-for-covid19-vaccines-what-you-need-to-know.
[12] 'NHS England » NHS Begins COVID-19 Booster Vaccination Campaign', accessed 18 November 2022, https://www.england.nhs.uk/2021/09/nhs-begins-covid-19-booster-vaccination-campaign/.
[13] 'NHS England » NHS to Begin Spring Booster Vaccinations This Week', accessed 18 November 2022, https://www.england.nhs.uk/2022/03/nhs-to-begin-spring-booster-vaccinations-this-week/.
[14] Met Office, 'UK Prepares for Historic Hot Spell', Met Office, 15 July 2022, https://www.metoffice.gov.uk/about-us/press-office/news/weather-and-climate/2022/red-extreme-heat-warning.
[15] NHS, 'Treatments for Coronavirus (COVID-19)', nhs.uk, 9 March 2022, https://www.nhs.uk/conditions/coronavirus-covid-19/self-care-and-treatments-for-coronavirus/treatments-for-coronavirus/.

a positive test prior to their presentation to ED are those more likely to have severe disease and be less representative of the general population. Conversely, those who have a test on the same day as their presentation to ED are likely to represent the general population, who have limited access to tests outside of healthcare settings. To account for this, a sensitivity analysis was restricted to the subset of the study population who attended ED the same day as their positive test. Results for this can be found in Supplementary Table S2.

To assess the impact of dissimilar numbers of cases and controls for each variant by calendar time, a sensitivity analysis was conducted restricted to those whose test dates were between 3 June to 13 June 2022, when the numbers with BA.2 or BA.4/5 were approximately equal. Results for this sensitivity analysis can be found in Supplementary Table S3.

Similarly, the impact of varying definitions of admission in outcome definition 1 was assessed. For this, all admissions with lengths of stay of 1 day or more were considered a valid admission; a separate outcome was also assessed where all admissions regardless of length of stay were considered a valid admission. Results for this can be found in Supplementary Table S4.

Regressions were also run for each covariate separately to assess which covariates have the greatest effect on the adjusted OR, the results of which can be found in Supplementary Table S5.

Data were linked, cleaned, and analysed using R version 4.2.2 and SQL Server 2019 (version 18.11).

Supplementary Tables

Supplementary Table S1. Descriptive statistics of the study population (n = 21,725) by variant type and outcome status based on outcome definition 1 where cases are individuals admitted for ≥2 days or died within 2 days of attending ED and controls are individuals discharged from ED or admitted for <2 days. Percentages are column-wise and describes the proportion of individuals in each subgroup.

	BA.2 (n = 9,804)		BA.4 (n = 2,042)		BA.5 (n = 9,879)	
	Cases (%) (n = 4,509)	Controls (%) (n = 5,295)	Cases (%) (n = 760)	Controls (%) (n = 1,282)	Cases (%) (n = 4,052)	Controls (%) (n = 5,827)
Age group						
0-9	133 (2.9)	390 (7.3)	34 (4.5)	148 (11.5)	159 (3.9)	562 (9.6)
10-19	40 (0.9)	170 (3.2)	11 (1.4)	59 (4.6)	34 (0.8)	162 (2.8)
20-29	111 (2.5)	460 (8.7)	16 (2.1)	138 (10.7)	93 (2.3)	528 (9.0)
30-39	128 (2.8)	577 (10.9)	31 (4.1)	142 (11.0)	145 (3.6)	573 (9.8)
40-49	160 (3.5)	460 (8.7)	36 (4.7)	123 (9.6)	185 (4.6)	494 (8.4)
50-59	301 (6.7)	576 (10.8)	73 (9.6)	139 (10.8)	305 (7.5)	688 (11.7)
60-69	555 (12.3)	645 (12.1)	93 (12.2)	161 (12.5)	534 (13.1)	748 (12.8)
70-79	1,129 (25.0)	836 (15.7)	179 (23.5)	179 (13.9)	1,002 (24.7)	944 (16.1)
80-89	1,380 (30.5)	882 (16.6)	207 (27.1)	137 (10.7)	1,171 (28.8)	854 (14.6)
≥ 90	584 (12.9)	319 (6.0)	83 (10.9)	60 (4.7)	435 (10.7)	304 (5.2)
Sex						
Female	2,175 (48.1)	2,880 (54.2)	381 (49.9)	714 (55.5)	1,998 (49.2)	3,044 (52.0)
Male	2,346 (51.9)	2,435 (45.8)	382 (50.1)	572 (44.5)	2,065 (50.8)	2,813 (48.0)
Vaccination status						
Unvaccinated	541 (12.0)	1,060 (19.9)	96 (12.6)	334 (26.0)	542 (13.3)	1,301 (22.2)
Dose 1	119 (2.6)	212 (4.0)	16 (2.1)	41 (3.2)	94 (2.3)	179 (3.1)
Dose 2	410 (9.1)	593 (11.2)	60 (7.9)	150 (11.7)	324 (8.0)	599 (10.2)
2/3 dose + early booster	3,130 (69.2)	3,174 (59.7)	396 (51.9)	569 (44.2)	1,909 (47.0)	2,680 (45.8)
2/3 dose + spring booster/2 boosters	321 (7.1)	276 (5.2)	195 (25.6)	192 (14.9)	1,194 (29.4)	1,098 (18.7)
Heatwave attendance status						
Not during heatwave	4,514 (99.8)	5,307 (99.8)	725 (95.0)	1,250 (97.2)	3,673 (90.4)	5,569 (95.1)
During heatwave	7 (0.2)	8 (0.2)	38 (5.0)	36 (2.8)	390 (9.6)	288 (4.9)
Prior infection status						
First infection	4,320 (95.6)	4,893 (92.1)	708 (92.8)	1,137 (88.4)	3,773 (92.9)	5,240 (89.5)
Reinfection	201 (4.4)	422 (7.9)	55 (7.2)	149 (11.6)	290 (7.1)	617 (10.5)
Index of multiple deprivation quintile						
1 (Most deprived)	1,076 (23.9)	1,260 (23.8)	170 (22.4)	309 (24.1)	977 (24.1)	1,407 (24.1)
2	965 (21.4)	1,175 (22.2)	163 (21.4)	268 (20.9)	795 (19.6)	1,261 (21.6)
3	886 (19.6)	1,001 (18.9)	145 (19.1)	254 (19.8)	838 (20.7)	1,151 (19.8)
4	834 (18.5)	1,018 (19.2)	132 (17.4)	261 (20.4)	772 (19.1)	1,065 (18.3)
5 (Least deprived)	748 (16.6)	841 (15.9)	150 (19.7)	190 (14.8)	670 (16.5)	943 (16.2)
Region						
London	642 (14.2)	761 (14.4)	100 (13.2)	146 (11.4)	512 (12.6)	703 (12.1)
Midlands	994 (22.0)	1,251 (23.6)	154 (20.3)	329 (25.7)	829 (20.5)	1,345 (23.1)
East of England	349 (7.7)	368 (6.9)	51 (6.7)	89 (6.9)	351 (8.7)	452 (7.8)
North East and Yorkshire	798 (17.7)	916 (17.3)	90 (11.8)	150 (11.7)	623 (15.4)	792 (13.6)
North West	818 (18.1)	784 (14.8)	159 (20.9)	210 (16.4)	697 (17.2)	865 (14.8)
South East	554 (12.3)	866 (16.4)	123 (16.2)	225 (17.6)	654 (16.1)	1,122 (19.3)

	BA.2 (n = 9,804)		BA.4 (n = 2,042)		BA.5 (n = 9,879)	
	Cases (%) (n = 4,509)	Controls (%) (n = 5,295)	Cases (%) (n = 760)	Controls (%) (n = 1,282)	Cases (%) (n = 4,052)	Controls (%) (n = 5,827)
South West	354 (7.9)	349 (6.6)	83 (10.9)	133 (10.4)	386 (9.5)	548 (9.4)

Supplementary Table S2. Crude and adjusted odds ratios comparing risk of admission or death among individuals who attended A&E the same day as their positive SARS-CoV-2 test for COVID-19 cases with Omicron BA.4 and BA.5 compared to Omicron BA.2, by outcome definition. Data includes positive tests in England between April – August 2022.

**Case Definition**	**BA.4 versus BA.2**							**BA.5 versus BA.2**						
	Sample Sizes for BA.4		Crude Model		Adjusted Stratified Model*			Sample Sizes for BA.5		Crude Model		Adjusted Stratified Model	
			OR (95% CI)	P-value	OR (95% CI)	P-value			OR (95% CI)	P-value	OR (95% CI)	P-value
Definition 1[†]	Cases	497	0.66 (0.59 - 0.75)	< 0.0001	0.91 (0.79 - 1.04)	0.178	Cases	2,558	0.77 (0.72 - 0.83)	< 0.0001	0.94 (0.84 - 1.06)	0.3061
	Controls	850					Controls	3,782				
Definition 2[‡]	Cases	77	0.79 (0.61 – 1.00)	0.0593	1.07 (0.73 - 1.55)	0.7339	Cases	413	0.91 (0.79 - 1.04)	0.1627	1.24 (0.9 - 1.7)	0.1895
	Controls	1,308					Controls	6,094				

* Model is a conditional logistic regression stratified by specimen test week, and adjusted for age group (10-year age bands), vaccination status, sex, prior infection status, and a flag for possible heatwave-related hospital attendances.

† Definition 1: A case is any individual whose ED attendance ended in hospital admission with a length of stay in hospital of 2 or more days; if their ED attendance ended in death; or if they had a date of death up to 2 days after their initial date of ED attendance. A control is any individual whose ED attendance ended with discharge or a hospital admission of less than 2 days' duration, and absence of death in the 2 days following ED attendance.

‡ Definition 2: A case is any individual who attended ED and received oxygen therapy in ED. A control is any individual who attended ED but did not receive oxygen therapy.

Supplementary Table S3. Crude and adjusted odds ratios using outcome definition 1† comparing risk of admission or death among individuals who attended A&E for COVID-19 cases with Omicron BA.4 and BA.5 compared to Omicron BA.2. Data includes positive tests in England between 06 June – 23 June 2022 when BA.4, BA.5 and BA.2 had comparable incidence.

**Model**	**BA.4 versus BA.2**		**BA.5 versus BA.2**	
	OR (95% CI)	P-value	OR (95% CI)	P-value
Crude model	0.59 (0.44 - 0.8)	0.0006	0.77 (0.61 - 0.98)	0.031
Adjusted model*	0.81 (0.64 - 1.04)	0.0938	0.94 (0.78 - 1.13)	0.4947

* Model is a conditional logistic regression stratified by specimen test week, and adjusted for age group (10-year age bands), vaccination status, sex, prior infection status, and a flag for possible heatwave-related hospital attendances.

† Definition 1: A case is any individual whose ED attendance ended in hospital admission with a length of stay in hospital of 2 or more days; if their ED attendance ended in death; or if they had a date of death up to 2 days after their initial date of ED attendance. A control is any individual whose ED attendance ended with discharge or a hospital admission of less than 2 days' duration, and absence of death in the 2 days following ED attendance.

Supplementary Table S4. Crude and adjusted odds ratios using outcome definition 1† comparing risk of admission or death among individuals who attended A&E for COVID-19 cases with Omicron BA.4 and BA.5 compared to Omicron BA.2, stratified by alternate admission definitions. Data includes positive tests in England between April – August 2022.

Admission Definition	BA.4 versus BA.2				BA.5 versus BA.2			
	Crude Model		Adjusted Stratified Model*		Crude Model		Adjusted Stratified Model	
	OR (95% CI)	P-value	OR (95% CI)	P-value	OR (95% CI)	P-value	OR (95% CI)	P-value
≥ 2 days length of stay (original definition)	0.7 (0.63 - 0.77)	< 0.0001	0.96 (0.86 - 1.08)	0.5036	0.82 (0.77 - 0.86)	< 0.0001	1.02 (0.93 - 1.12)	0.6298
≥ 1 day length of stay	0.78 (0.71 - 0.86)	< 0.0001	0.95 (0.86 - 1.04)	0.2692	0.87 (0.82 - 0.92)	< 0.0001	0.97 (0.89 - 1.05)	0.4009
No length of stay cut-off	0.79 (0.72 - 0.87)	< 0.0001	0.94 (0.86 - 1.04)	0.223	0.91 (0.86 - 0.97)	0.0015	0.97 (0.9 - 1.05)	0.4334

* Model is a conditional logistic regression stratified by specimen test week, and adjusted for age group (10-year age bands), vaccination status, sex, prior infection status, and a flag for possible heatwave-related hospital attendances.
† Definition 1: A case is any individual whose ED attendance ended in hospital admission with a length of stay in hospital of 2 or more days; if their ED attendance ended in death; or if they had a date of death up to 2 days after their initial date of ED attendance. A control is any individual whose ED attendance ended with discharge or a hospital admission of less than 2 days' duration, and absence of death in the 2 days following ED attendance.

Supplementary Table S5. Odds ratios using one-at-a-time logistic regression to assess covariate effects using outcome definition 1†. Crude model compares risk of admission or death among individuals who attended A&E for COVID-19 cases with Omicron BA.4 and BA.5 compared to Omicron BA.2. Data includes positive tests in England between April – August 2022.

Model	BA.4 versus BA.2			BA.5 versus BA.2		
	Odds Ratio (95% CI)	Difference from crude OR	P-value	Odds Ratio (95% CI)	Difference from crude OR	P-value
Crude model	0.76 (0.7 - 0.82)	0.00	< 0.0001	0.86 (0.82 - 0.9)	0.00	< 0.0001
Crude model + age group	0.86 (0.79 - 0.92)	0.10	< 0.0001	0.89 (0.86 - 0.93)	0.03	< 0.0001
Crude model + heatwave admission	0.75 (0.69 - 0.81)	-0.01	< 0.0001	0.83 (0.79 - 0.87)	-0.03	< 0.0001
Crude model + IMD quintile	0.76 (0.7 - 0.82)	0.00	< 0.0001	0.86 (0.82 - 0.9)	0.00	< 0.0001
Crude model + region	0.76 (0.7 - 0.82)	0.00	< 0.0001	0.86 (0.83 - 0.9)	0.00	< 0.0001
Crude model + reinfection status	0.77 (0.71 - 0.83)	0.01	< 0.0001	0.87 (0.83 - 0.91)	0.01	< 0.0001
Crude model + sex	0.76 (0.71 - 0.82)	0.00	< 0.0001	0.86 (0.82 - 0.9)	0.00	< 0.0001
Crude model + stratified test week	0.85 (0.76 - 0.95)	0.09	0.0045	0.95 (0.87 - 1.04)	0.09	0.2451
Crude model + vaccination status	0.76 (0.7 - 0.82)	0.00	< 0.0001	0.83 (0.79 - 0.86)	-0.03	< 0.0001

† Definition 1: A case is any individual whose ED attendance ended in hospital admission with a length of stay in hospital of 2 or more days; if their ED attendance ended in death; or if they had a date of death up to 2 days after their initial date of ED attendance. A control is any individual whose ED attendance ended with discharge or a hospital admission of less than 2 days' duration, and absence of death in the 2 days following ED attendance.
